# Effects of Different Live-Preservation Methods on Soft-Shell Hardening and Flavor Characteristics of the Mud Crab (*Scylla paramamosain*)

**DOI:** 10.3390/foods15020344

**Published:** 2026-01-17

**Authors:** Ye Sun, Shengming Han, Yangfang Ye

**Affiliations:** 1 Key Laboratory of Aquacultural Biotechnology of the Ministry of Education, School of Marine Sciences, Ningbo University, Ningbo 315832, China; 2 Ningbo Dasheng Biotechnology Co., Ltd., Ningbo 315708, China

**Keywords:** soft-shell crab, shell hardening, flavor, ice-chilled storage, transcriptomic analysis, low pH

## Abstract

The soft-shell mud crab (*Scylla paramamosain*) holds high market value, but rapid post-molting shell hardening limits its commercial viability. This study evaluated the effects of four live-preservation methods—ambient seawater (CK, 25 °C, pH 8.10), low-temperature seawater (LT, 14 °C, pH 8.10), ice-chilled storage (ICE, 2–6 °C), and low-temperature acidified seawater (LTA, 14 °C, pH 7.6)—on shell hardening and hepatopancreatic flavor in mud crabs. ICE and LTA significantly delayed hardening (*p* < 0.05), maintaining the hard-paper stage at 48 h post-molting, while CK and LT samples hardened considerably. Transcriptomic analysis revealed that both ICE and LTA down-regulated key genes involved in calcium signaling, autophagy, and lysosomal pathways, which may be associated with delayed shell hardening. Flavor profiling showed that ICE enhanced umami by increasing aspartate, inosine monophosphate, and adenosine monophosphate levels, and increased sweetness via elevated alanine and glycine, while reducing bitterness by lowering bitter amino acids. In contrast, LTA reduced umami and bitterness but did not improve sweetness. These findings demonstrate that ice-chilled storage effectively extends the soft-shell phase and better preserves flavor quality, offering a viable strategy for enhancing the preservation and marketability of live soft-shell crabs.

## 1. Introduction

The mud crab (*Scylla paramamosain*) is a valuable marine crab species of significant economic importance in China. However, its hard shell and aggressive nature make consumption inconvenient. In contrast, soft-shell crabs—harvested during the molting period before shell hardening—are not only easier to eat but also rich in calcium and low in fat. As a result, they command a market price up to seven times higher than that of hard-shell crabs [1], positioning them as one of the most promising varieties commercially [2]. China leads global soft-shell crab production, primarily farming *S. paramamosain*, and supplies live, frozen, and processed products [3]. Among these, live soft-shell crabs are considered the premium choice for consumption. Nevertheless, after molting, crabs rapidly absorb Ca^2+^, CO_2_, and HCO_3_^−^ from seawater to synthesize and deposit CaCO_3_ onto the protein matrix of the new exoskeleton, leading to full hardening within about two days. The high-value window for soft-shell crabs is even narrower, averaging only about 3 h [4,5,6]. Thus, extending the soft-shell stage is a critical challenge for the industry.

Previous studies suggest that low temperatures can reduce the metabolic rate and energy consumption in mud crabs [7], potentially delaying shell calcification. However, the direct effect of low temperature on soft-shell hardening remains unexplored. Our earlier work indicates that lower seawater pH can delay shell hardening [8], likely by reducing CaCO_3_ saturation and inhibiting mineralization. Supporting this, research on blue crabs showed that maintaining water pH below 7.3 and total ammonia above 6.0 mg/L could prolong the soft-shell stage for up to five days [9]. These findings highlight the need to systematically study the combined effects of low temperature and low pH on soft-shell hardening in *S. paramamosain*.

As an aquatic product, the flavor profile of soft-shell crabs determines their sensory characteristics. Flavor compounds are categorized into non-volatile and volatile types. Non-volatile components—especially nitrogen-free and nitrogen-containing compounds—play a key role in taste and serve as precursors for volatile aromas [10]. In crabs, free amino acids (FAAs) and nucleotides are particularly influential [11,12]. FAAs contribute distinct taste qualities—such as umami, sweetness, and bitterness—and their combination forms the characteristic flavor profile. In particular, umami amino acids (UAAs) like glutamate (Glu) and aspartate (Asp) act synergistically with flavor nucleotides such as adenosine monophosphate (AMP), inosine monophosphate (IMP), and guanosine monophosphate (GMP), markedly enhancing overall umami perception [13,14]. Sweetness, another key flavor attribute in crabs, is primarily imparted by sweet amino acids (SAAs) like serine (Ser), threonine (Thr), glycine (Gly), alanine (Ala), and proline (Pro) [15]. In *S. paramamosain*, alongside umami and sweet tastes, the hepatopancreas also presents a mild bitter taste, largely due to bitter amino acids (BAAs) such as arginine (Arg), tryptophan (Trp), tyrosine (Tyr), and lysine (Lys) [16]. Although Arg contributes bitterness, it can also elevate umami perception and help balance off-flavors like fishiness [17].

In this study, we examined how low-temperature and low-pH live-preservation methods affect shell hardening and related gene expression in the hepatopancreas of *S. paramamosain*. We also analyzed their impacts on FAAs and flavor nucleotides in the hepatopancreas. The aim is to assess live-preservation approaches from both hardening-delay and flavor-quality perspectives, providing insights for developing effective strategies to maintain soft-shell crabs in live condition.

## 2. Materials and Methods

### 2.1. Soft-Shell Crab Production and Sample Collection

A total of 100 live hard-shell mud crabs (approximately 150 g each) were purchased from a local aquatic product trading market in Sanmen, Zhejiang, China and transported to the laboratory within two hours in insulated foam boxes under dry conditions at an ambient temperature of approximately 25 °C. Each crab was transferred into a perforated plastic basket (320 mm × 190 mm × 170 mm) and was then randomly distributed to one of 10 canvas tanks (1.5 m × 1.0 m × 0.9 m), each filled with 300 L of natural seawater. The rearing water was continuously aerated, with aerated water parameters maintained as follows: temperature 25 °C, salinity 25 PSU, pH 8.10, dissolved oxygen > 5 mg/L, and ammonia nitrogen <0.50 mg/L. A 30% daily water exchange was performed, and no feeding was conducted throughout the rearing period. Four live-preservation methods were implemented in this study: (1) Crabs held in seawater at 25 °C (control group, CK); (2) Crabs held in seawater precooled to 14 °C using a chiller (Guangli Cooling Equipment Company, Guangzhou, China) (low-temperature group, LT); (3) Crabs placed in a small polystyrene box (320 mm × 190 mm × 170 mm) nested within a larger one (390 mm × 280 mm × 200 mm) with crushed ice between the boxes and covered with a moist towel (ice-chilled group, ICE; air temperature approximately 2–6 °C); and (4) Crabs held in seawater at 14 °C with pH adjusted to 7.60 using 99.5% acetic acid (Sigma-Aldrich, St. Louis, MO, USA) (low-temperature acidified group, LTA). For subsequent analysis, sample designations combine the group abbreviation with the post-molting time point (in hours), e.g., CK48 refers to the control group at 48 h. Crab molting was closely monitored, and 24 newly molted crabs were collected and evenly distributed among the four experimental groups, with six crabs per group.

Shell hardness was evaluated at 0, 12, 24, and 48 h after molting using the standardized tactile scale described by Hungria et al. [9], which classifies exoskeleton hardness into five grades: soft, leathery, soft paper, hard paper, and hard. All measurements were conducted by a single trained researcher to maintain consistency. The shell hardness of all 24 crabs was assessed at each designated time point. At 48 h post-molting, the crabs were anesthetized in ice-cold water, and hepatopancreas tissue was collected. The samples were immediately frozen in liquid nitrogen and stored at −80 °C until analysis. The hepatopancreas was selected for this study because it is both a preferred edible tissue for consumers and the primary metabolic organ in crabs.

### 2.2. Bulk RNA-Seq and Bioinformatic Analysis

Hepatopancreas samples from six crabs per group were used for transcriptomic analysis. Total RNA was extracted using TRIzol reagent (Invitrogen, Carlsbad, CA, USA), and RNA integrity was confirmed (RIN ≥ 8). Sequencing libraries were constructed from 1 μg of high-quality RNA using the Illumina Stranded mRNA Prep kit (Illumina Inc., San Diego, CA, USA) and subsequently sequenced on an Illumina NovaSeq 6000 platform (Illumina Inc., San Diego, CA, USA) to generate 150 bp paired-end reads.

Raw reads were quality-controlled using fastp (version 0.23.2) [18]. Clean reads were aligned to the reference genome using HISAT2 (version 2.2.1) [19] and assembled with StringTie (version 2.2.0) [20]. Gene expression levels were quantified using RSEM [21], and differential expression analysis was performed with DESeq2 [22] under the thresholds |log_2_FC| ≥ 1 and FDR ≤ 0.05.

### 2.3. Ultra-Performance Liquid Chromatography-Tandem Mass Spectrometry (UPLC-MS/MS) Analysis of FAAs

Frozen hepatopancreas samples (20 mg) were homogenized in 470 μL of ice-cold methanol-water extraction solution (2:1, *v*/*v*; HPLC-grade methanol, Merck KGaA, Darmstadt, Germany) using a cryogenic tissue grinder (JXFSTPRP-CLN series, Jingxin, Shanghai, China) at 50 Hz for 90 s. The homogenate was centrifuged at 13,700× *g* for 10 min at 4 °C (Centrifuge 5424 R, Eppendorf, Hamburg, Germany), and the supernatant was collected. The residual pellet was re-extracted following the same procedure, and the resulting supernatant was pooled with the first extract. The combined supernatant was derivatized by sequential addition of: (1) N-ethylmaleimide-containing phosphate buffer, (2) 0.23 mol/L dimethyl sulfoxide, (3) borate buffer, and (4) 5-aminoisoquinoline-1-carbonyl cyanide derivatization reagent dissolved in acetonitrile (HPLC-grade, Merck KGaA, Darmstadt, Germany). After each addition, the mixture was vortexed and then incubated at 55 °C for 10 min. The derivatization reaction was quenched with formic acid. Following centrifugation, the supernatant was filtered through a 0.22 μm membrane filter, and the resulting filtrate was used for chromatographic analysis.

Chromatographic separation and mass spectrometric detection were performed on an Agilent 1290 UPLC system coupled to an Agilent 6470 triple-quadrupole mass spectrometer (Agilent Technologies, Santa Clara, CA, USA). Separation was achieved on an Agilent ZORBAX Eclipse Plus C18 column (2.1 × 100 mm, 1.8 μm; Agilent Technologies) maintained at 35 °C. Mobile phase A consisted of ultrapure water containing 0.004% formic acid and 5 mM ammonium bicarbonate; mobile phase B was methanol containing 0.16% formic acid and 2 mM ammonium formate. Mass detection was performed in positive electrospray ionization mode with multiple reaction monitoring, using optimized instrument parameters. Data were processed and quantified with Mass Hunter Workstation software (version B.08.00, Agilent Technologies). Statistical differences between groups were evaluated using the two-tailed Wilcoxon rank-sum test.

### 2.4. Ultra Performance Liquid Chromatography (HPLC) Analysis of Flavor Nucleotides

The hepatopancreas sample (250 mg) was homogenized in 1.5 mL of 10% perchloric acid, followed by sonication on wet ice using an ultrasonic cleaner (Branson Ultrasonics, Brookfield, CT, USA) at 30% amplitude for a total of 3 min (applied in three 60 s pulses with 30 s intervals to prevent overheating). After centrifugation (4 °C, 13,700× *g*, 15 min; Centrifuge 5424 R, Eppendorf, Hamburg, Germany), the pellet was re-extracted using the same procedure. The resulting supernatants were combined, diluted to 5 mL with 0.05 M phosphoric acid in methanol-water (5:95, *v*/*v*), filtered through a 0.22 μm membrane, and subjected to HPLC analysis.

The analysis was conducted on an Agilent 1260 HPLC system (Agilent Technologies) equipped with a Shim-pack Gis C18 column (Shimadzu Corporation, Kyoto, Japan). Separation was performed using a gradient elution with methanol (mobile phase A) and phosphate buffer (pH 5.35, mobile phase B). Detection was carried out at 254 nm, and the column temperature was maintained at 30 °C. Quantification was based on retention time and calibration curves (R^2^ > 0.99). All samples were analyzed in triplicate.

### 2.5. Taste Activity Value (TAV) Calculation

TAV was calculated as TAV = C/T, where C is the concentration of the flavor compound (mg/100 g) and T is its sensory threshold in the corresponding matrix. A TAV ≥ 1 indicates a significant contribution to flavor, with higher values representing greater influence [23].

### 2.6. Equivalent Umami Concentration (EUC) Calculation

To quantify synergistic umami intensity, EUC was calculated using the model by Yamaguchi et al. [24]: EUC = ∑a_i_b_i_ + 1218(∑a_i_b_i_)(∑a_j_b_j_). In this formula, a_i_ and a_j_ were concentrations (g/100 g) of Glu, Asp, IMP, GMP, and AMP, respectively, b_i_ and b_j_ are their relative umami coefficients (Glu: 1, Asp: 0.077; IMP: 1, GMP: 2.3, AMP: 0.18), and 1218 was the synergistic constant. Higher EUC values indicate stronger umami synergy.

### 2.7. Statistical Analyses

Data are presented as the mean ± standard deviation (SD) (*n* = 6). Differences in flavor nucleotides and EUC between groups were evaluated by the Kruskal–Wallis test with Benjamini–Hochberg *p*-value correction. Differences in FAA data were analyzed by one-way ANOVA followed by Duncan’s multiple range test for post hoc comparisons. All statistical analyses were performed using R software (version 4.4.0), with the tidyverse package suite utilized for data handling and visualization. Differences were considered statistically significant at *p* < 0.05.

## 3. Results

### 3.1. Impact of Live-Preservation Methods on Shell Hardening

**Figure 1:**
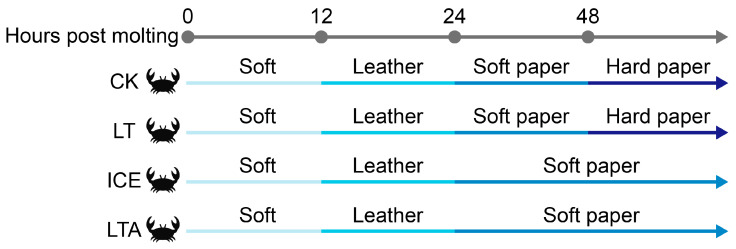
Effects of different live-preservation methods on the shell hardening of mud crabs.

The shell hardening process differed across the live-preservation methods. In the CK group, crab shells reached the leathery stage at 12 h, the soft paper stage by 24 h, and the hard paper stage after 48 h (Figure 1). Crabs in the LT group exhibited a similar hardening progression, also attaining the hard paper stage by 48 h. In contrast, both the ICE and LTA crabs showed delayed shell hardening, with shells remaining at the soft paper stage even at 48 h post-molting. The visual progression of this delayed hardening has been recorded in a related study [25].

### 3.2. Impact of Live-Preservation Methods on Gene Transcriptional Expressions of Hepatopancreas

**Figure 2:**
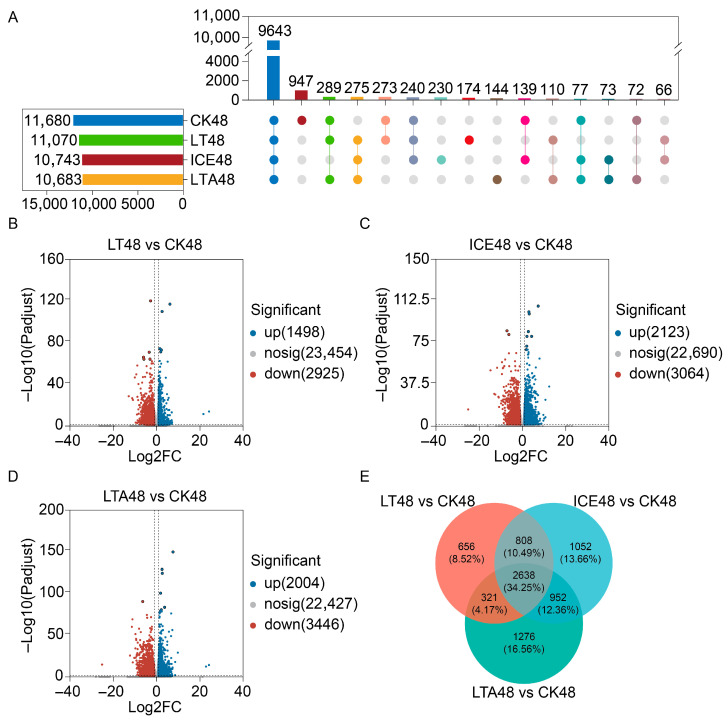
Analysis of differential gene expression in the hepatopancreas of mud crabs under different preservation conditions. (**A**) Upset plot illustrating the number of shared and unique genes across treatment groups. (**B**) Bar plot of differentially expressed genes (DEGs) in the LT48 group compared with the CK48 group. (**C**) Bar plot of DEGs in the ICE48 group compared with the CK48 group. (**D**) Bar plot of DEGs in the LTA48 group compared with the CK48 group. (**E**) Venn diagram depicting the shared DEGs by different live-preservation methods.

Upset plot analysis of hepatopancreas transcriptomes at 48 h post-molting revealed distinct gene expression profiles among groups (Figure 2A). A total of 11,680, 11,070, 10,743, and 10,683 genes were detected in the hepatopancreas samples of CK48, LT48, ICE48, and LTA48 crabs, respectively. Of these, 9643 genes were expressed in all groups, while 947, 174, 230, and 144 genes were specifically expressed in the CK48, LT48, ICE48, and LTA48 groups, respectively. Differential expression analysis further highlighted distinct transcriptional responses across live-preservation methods. Compared to the CK48 group, the hepatopancreas of LT48 crabs exhibited 4423 (1498 up-regulated, 2925 down-regulated; Figure 2B). ICE48 crabs showed 5187 DEGs (2123 up-regulated, 3064 down-regulated; Figure 2C), and LTA48 crabs displayed 5450 DEGs (2004 up-regulated, 3446 down-regulated; Figure 2D) relative to CK48. Venn diagram analysis further revealed overlaps in DEGs between groups: 321 DEGs were shared between LT48 and ICE48, 808 between LT48 and LTA48, and 952 between ICE48 and LTA48 (Figure 2E).

**Figure 3:**
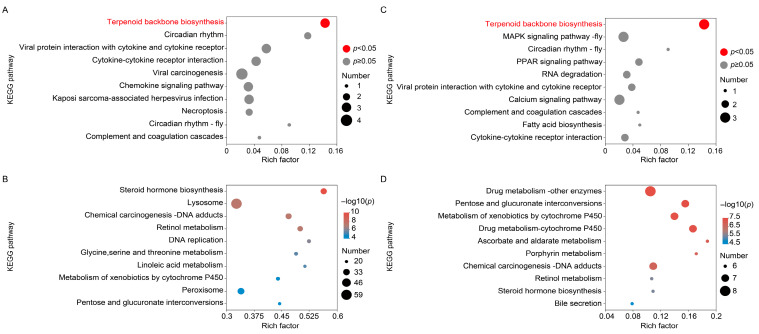
Functional enrichment analysis of differentially expressed genes (DEGs) in the ICE48 and LTA48 crabs. (**A**) Significantly enriched KEGG pathway for up-regulated DEGs in the ICE48 crabs. (**B**) Top 10 significantly enriched KEGG pathways for down-regulated DEGs in the ICE48 crabs. (**C**) Significantly enriched KEGG pathway for up-regulated DEGs in the LTA48 crabs. (**D**) Top 10 significantly enriched KEGG pathways for down-regulated DEGs in the LTA48 crabs.

To elucidate the molecular functional changes associated with delayed shell hardening, functional enrichment analysis was conducted on DEGs between the ICE48/LTA48 groups and CK48 control. Results revealed that in the ICE48 group, the up-regulated DEGs were significantly enriched in terpenoid backbone biosynthesis (Figure 3A), while down-regulated DEGs were predominantly enriched in steroid hormone biosynthesis, lysosome, and glycine, serine, and threonine metabolism (Figure 3B). Similarly, in the LTA48 group, up-regulated DEGs were also enriched in terpenoid backbone biosynthesis (Figure 3C). In contrast, the down-regulated DEGs in LTA48 were mainly associated with drug metabolism-other enzymes, retinol metabolism, and metabolism of xenobiotics by cytochrome P450, but not with steroid hormone biosynthesis (Figure 3D).

**Figure 4:**
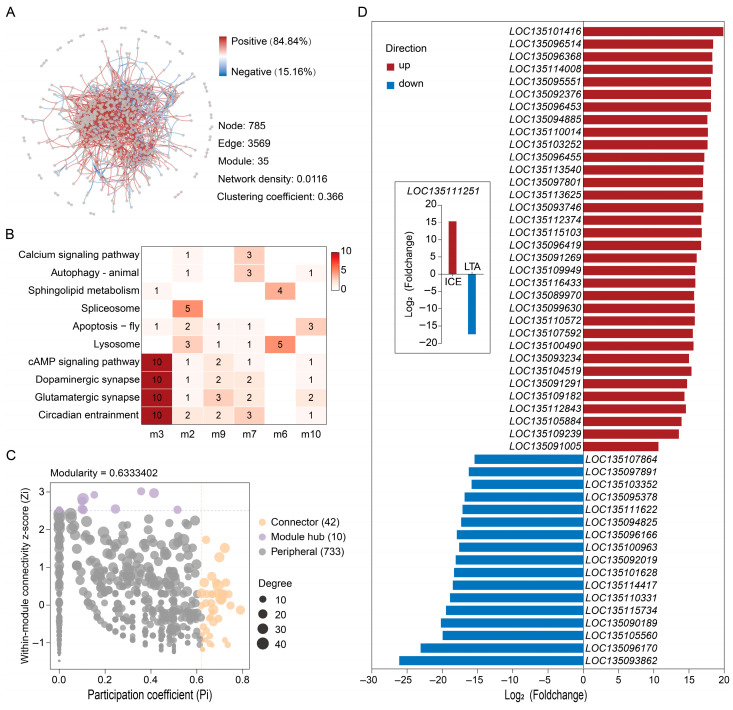
Co-expression network analysis of DEGs shared by ICE48 and LTA48 crabs. (**A**) Global topology of the network filtered by high-confidence interactions (|r| > 0.8, q < 0.05). (**B**) Functional distribution heatmap of filtered high-quality modules, showing the number of genes associated with significantly enriched pathways (filtering criteria: ≥10 annotated genes, ≥50% annotation rate, ≥3 distinct functions, and ≥3 genes for the top function). (**C**) Identification of hub genes using defined topological thresholds (Z_i_ > 2.5, P_i_ > 0.62). (**D**) Expression patterns of the 52 key hub genes, showing coordinated regulation with a single exception.

Given that both ICE48 and LTA48 groups exhibited delayed soft-shell hardening, a co-expression network was constructed using the 952 DEGs shared by these two groups. The resulting network contained 785 nodes and 3569 edges, and was partitioned into 35 modules characterized by low density, a high clustering coefficient, and predominantly positive correlations (Figure 4A). Functional enrichment analysis of these modules showed significant enrichment in key pathways associated with shell hardening and environmental stress, including the calcium signaling pathway, autophagy, lysosome, and sphingolipid metabolism. Within the network, 52 genes were identified as key nodes, including 10 module hubs and 42 connectors (Figure 4C). Notably, several hub genes were functionally annotated to pathways critical for shell formation and cellular remodeling. For instance, *LOC135094825* was linked to the calcium signaling pathway, *LOC135095378* to lysosome pathway, and *LOC135094885* to autophagy and mitophagy (Supplementary Table S1). The expression patterns of these 52 key genes were highly coordinated between the ICE and LTA groups, with 51 genes showing consistent up- or down-regulation in both conditions (Figure 4D). Only one gene displayed opposing expression trends, being up-regulated in ICE and down-regulated in LTA.

### 3.3. Impact of Live-Preservation Methods on the Flavor-Related Compounds in the Hepatopancreas

To assess whether ICE48 and LTA48 crabs—which exhibited softer shells—possessed improved taste compared to CK48, FAAs and flavor nucleotides in the hepatopancreas were further analyzed. A total of 20 FAAs were identified, comprising two UAAs, five SAAs, 10 BAAs, and three tasteless amino acids (Table 1). In CK48 crabs, the predominant flavor-related amino acids—Glu, Ala, Gly, Arg, Lys, leucine (Leu), and phenylalanine (Phe)—each exceeded 100 mg/100 g. Notably, Gly and Leu had TAVs below 1, whereas valine (Val), histidine (His), and methionine (Met), despite their lower contents (<100 mg/100 g), displayed TAVs above 1. FAA profiles differed markedly among live-preservation methods. Relative to CK48, ICE48 crabs showed significantly higher levels of Asp, Ala, and Gly, but lower levels of the other three SAAs and nearly all BAAs except Arg (*p* < 0.05). Correspondingly, the TAV of Ala rose from 4.16 to 9.00, and that of Gly increased from 0.92 to 1.06. In contrast, the TAV of Lys decreased from 2.36 to 1.39, and the TAVs of Phe, Val, His, and Met all fell below 1. Relative to CK48, LTA48 crabs exhibited significantly elevated Asp and Ala, but reduced Glu, Pro, Ser, Thr, and all BAAs (*p* < 0.05). The TAV of Ala increased from 4.16 to 5.47, while that of Glu decreased from 4.77 to 2.73. The TAVs of Arg and Lys declined from 8.53 and 2.36 to 5.47 and 1.96, respectively, and the TAVs of Phe, Val, His, and Met all dropped below 1. These alterations in FAA composition led to significant changes in the total amino acid (TAA), essential amino acid (EAA), UAA, SAA, and BAA contents. The TAA content was significantly lower in ICE48 (1555.35 mg/100 g) and LTA48 (1226.27 mg/100 g) than in CK48 (1957.65 mg/100 g) (*p* < 0.05). Similarly, EAA content was significantly reduced in ICE48 (148.41 mg/100 g) and LTA48 (220.86 mg/100 g) compared to CK48 (553.00 mg/100 g) (*p* < 0.05). The UAA content was significantly higher in ICE48 (213.06 mg/100 g) than in CK48 (168.92 mg/100 g), but lower in LTA48 (152.83 mg/100 g) (*p* < 0.05). The SAA content followed a trend similar to that of UAA. Moreover, BAA content was significantly lower in both ICE48 (567.58 mg/100 g) and LTA48 (547.36 mg/100 g) than in CK48 (1 090.45 mg/100 g) (*p* < 0.05).

**Figure 5:**
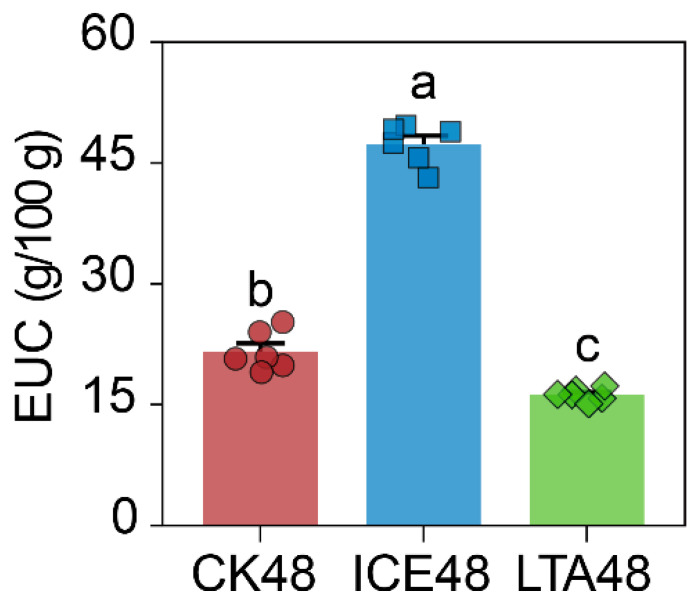
Equivalent umami concentration (EUC) in the hepatopancreas of soft-shell crabs under different storage conditions. Different letters above bars indicate significant differences among groups (*p* < 0.05).

Moreover, three flavor nucleotides—IMP, AMP, and GMP—were quantified in the hepatopancreas of soft-shell crabs. In the CK48 group, IMP was the predominant flavor nucleotide (91.79 mg/100 g), followed by AMP (43.22 mg/100 g) and GMP (9.46 mg/100 g) (Table 2). Among these, only IMP exhibited a TAV greater than one. The levels of all three nucleotides varied significantly across live-preservation methods. Compared to CK48, the ICE48 group showed significantly increased contents of IMP, AMP, and GMP (*p* < 0.05). The IMP content rose to 233.12 mg/100 g, accompanied by an increase in its TAV from 3.67 to 9.32. Notably, the TAV of AMP increased from 0.86 to 1.07. In the LTA48 group, IMP and AMP contents were significantly higher than those in CK48 (*p* < 0.05). In contrast to the ICE48 group, AMP was the most abundant nucleotide in LTA48 (122.79 mg/100 g), followed by IMP (106.32 mg/100 g). Accordingly, the TAV of AMP increased from 0.86 to 2.46, and that of IMP rose from 3.67 to 4.25. These changes resulted in a significantly higher total flavor nucleotide content in both the ICE48 (298.38 mg/100 g) and LTA48 (239.35 mg/100 g) groups compared to CK48 (144.46 mg/100 g) (*p* < 0.05). Quantitative analysis of EUC revealed that the EUC was significantly higher in the ICE48 group (47.37 g/100 g) but significantly lower in the LTA48 group (16.24 g/100 g) compared to the CK48 group (21.60 g/100 g) (*p* < 0.05, Figure 5).

## 4. Discussion

After molting, crabs enter a soft-shell stage, during which the new exoskeleton hardens through a process of flexible cuticular sclerotization and mineralization [26]. Mineralization primarily involves the deposition of CaCO_3_ around the organic matrix of the newly formed exoskeleton [27,28]. The formation of CaCO_3_ requires a supply of Ca^2+^ and CO_3_^2−^. Calcium ions are transported from the hemolymph to the exoskeleton via various calcium transporters located in epidermal cell membranes, while carbonate ions are supplied through the reversible hydration of CO_2_ to HCO_3_^−^, catalyzed by membrane-bound carbonic anhydrases (CAs) [29,30,31]. The direction of this chemical equilibrium is pH-dependent, with acidic conditions favoring the formation of CO_2_. In this study, the reduced seawater pH in the LTA treatment (pH 7.6) likely shifts the carbonate equilibrium toward CO_2_, thereby limiting the local availability of HCO_3_^−^ and CO_3_^2−^ at the calcification front. This carbonate substrate limitation likely contributes to the delayed shell hardening observed in the LTA group. In contrast, the ICE treatment appears to slow hardening mainly by reducing metabolic activity and ion absorption in crabs under low-temperature conditions, without substantially altering the pH-dependent carbonate equilibrium.

Transcriptomic analysis of the hepatopancreas under different live-preservation conditions revealed DEGs spanning multiple functional categories relevant to exoskeleton formation, including cuticle protein synthesis, ecdysteroid signaling, mineral ion transport, chitin metabolism, and calcium signaling (Supplementary Table S2). Although none of these pathways showed statistically significant enrichment in our analysis, A co-expression network analysis of shared DEGs from the ICE48 and LTA48 groups identified a core transcriptional program associated with delayed shell hardening, centered on the suppression of biomineralization and cellular remodeling. The highly synchronized expression of 51 key genes points to conserved molecular mechanisms under both preservation conditions. Functional enrichment analysis revealed significant downregulation of the calcium signaling pathway, which is essential for Ca^2+^ mobilization and transport—both prerequisites for the synthesis and deposition of CaCO_3_ onto the newly formed protein matrix [32,33,34]. This downregulation directly links the low-temperature and acidified preservation environments to the delayed hardening phenotype. Concurrently, pathways involved in cellular remodeling were inhibited, including lysosomal activity, which is necessary for degrading the old cuticle and recycling components to support new cuticle assembly during molting [35]. Similarly, suppression of steroid hormone biosynthesis disrupted the hormonal regulation of the molting cycle and associated energy metabolism [36,37]. Thus, our transcriptional data suggest that coordinated downregulation of calcium signaling, lysosomal function, and steroid hormone-mediated processes collectively underlies the delayed hardening observed in both groups. It should be noted, however, that the hepatopancreas serves primarily as a metabolic organ, and its transcriptional profile reflects systemic stress responses rather than specifically capturing local events at the calcification front (epidermis/exoskeleton). The tissue source of RNA is therefore a key factor limiting the specificity of our enrichment analysis. Future studies employing direct sampling of epidermal or calcifying matrix tissue during the hardening process would yield a more precise transcriptomic profile of delayed biomineralization under different live-preservation conditions. In addition to lowering seawater pH, the acetic acid introduced in the LTA treatment may also adversely affect shell calcification by reacting with CaCO_3_ already deposited in the shell. This likely represents another contributing factor to the delayed hardening of soft-shell crabs in the LTA group.

Umami and sweetness are critical quality attributes in aquatic products, largely determined by FAA and flavor nucleotides [38]. The hepatopancreas of ICE48 crabs exhibited a higher level of UAA relative to CK48, enhancing the umami intensity. Glu and Asp are the primary contributors to umami taste [39]. In ICE48 crabs, Glu maintains a TAV ≥ 1, indicating its dominant role in umami perception. Although Asp had a TAV below 1, its content was higher in ICE48 than in CK48, suggesting that it also contributed to the increasing of UAA in the hepatopancreas. In contrast, LTA48 crabs presented significantly lower UAA value, reflecting inferior umami quality, which can be attributed largely to a marked reduction in Glu content.

Among SAAs, Ala and Gly are particularly important [40,41]. Ala was the most abundant SAA in mud crab hepatopancreas, and its content was further elevated in ICE48 samples. Given its high concentration and low taste threshold (60), Ala served as a major contributor to sweetness, especially under ice-chilled storage. Gly was the second most abundant SAA; despite its higher taste threshold (130) and lower content relative to Ala, ice-chilled storage increased its contribution to sweetness. Gly also moderates bitterness, thereby improving overall taste acceptability. Other SAAs, including Pro, Ser, and Thr, decreased under ice-chilled storage. Due to their high thresholds, however, their influence on sweetness was limited. In LTA48 crabs, total SAA levels were significantly lower than in the control, yet Ala content was notably higher. Given Ala’s strong influence on sweetness, the hepatopancreas of LTA48 crabs likely presents a sweeter taste than that of the control crabs.

In contrast to UAAs and SAAs, BAAs levels were significantly lower in ICE48 crabs compared to CK48. Arg was the most abundant BAA, and its content was further reduced in ICE48 samples. Other key BAAs such as Lys, His, Val, Phe, and Met also showed significantly decreased contents and lower TAVs, collectively helping to minimize perceived bitterness. While moderate bitterness can add flavor complexity [42], excessive bitterness is generally undesirable. A similar reduction in BAA content was observed in LTA48 samples, indicating attenuated bitterness in the hepatopancreas of crabs from this group.

Notably, both ICE48 and LTA48 crabs exhibited significantly lower levels of EAAs in the hepatopancreas. EAAs—including Val, Leu, Ile, Phe, Trp, His, Met, Thr, and Lys—cannot be synthesized de novo and must be obtained from the diet. Their depletion under low-temperature and acidified conditions likely reflects a dual physiological role: serving as a nutritional reservoir and acting as signaling molecules in stress response. However, the reduction in EAA content inevitably diminishes the nutritional value of soft-shell crabs.

Flavor nucleotides, particularly AMP, IMP, and GMP, are also key taste determinants in crustaceans [43]. IMP, which degrades slowly post-harvest, accumulates in fresh products and serves as a key umami nucleotide [44]. In mud crab hepatopancreas, IMP was the abundant flavor nucleotide in the hepatopancreas of mud crabs. ice-chilled storage further increased IMP content, yielding a TAV nearly double that of Glu, thereby establishing IMP as the dominant umami contributor. IMP also acts synergistically with Glu to enhance umami perception [45]. Although less abundant, AMP reached a TAV > 1 under ice-chilled storage, indicating a noticeable umami contribution. GMP content remained low, with TAV < 1 even under ice-chilled storage, suggesting a minor—though potentially synergistic—role in flavor enhancement [46]. Low-temperature acidified seawater also significantly increased total nucleotide content, but with AMP exceeding IMP, leading to a greater relative contribution of AMP to umami. Despite this, ice-chilled storage resulted in a significantly higher EUC value, whereas low-temperature acidified seawater treatment produced the opposite trend.

Transcriptomic differences between the two storage conditions help explain their divergent flavor outcomes. Ice-chilled storage selectively enhanced desirable flavors by downregulating specific metabolic pathways—such as glycine, serine, and threonine metabolism—thereby reshaping the amino acid pool toward a sweeter, less bitter profile. In contrast, low-temperature acidified seawater broadly suppressed metabolic activity, precluding such targeted flavor modulation and resulting in an inferior sensory profile. Thus, from a flavor perspective, ice-chilled storage appears superior to low-temperature acidified seawater storage.

It is noteworthy that the hepatopancreas of both ICE48 and LTA48 crabs exhibited significantly reduced levels of EAAs. EAAs refer to the nine amino acids that cannot be synthesized de novo by the body and must be obtained from dietary sources, including Val, Leu, Ile, Phe, Trp, His, Met, Thr, and Lys [47]. The observed depletion of EAAs likely indicates their dual role as a nutritional resource and as a signaling regulator [48,49] in response to adverse environmental conditions—specifically, low temperature and acidification in this study. However, the reduction in EAA content in the hepatopancreas of ICE48 and LTA48 crabs inevitably diminishes the nutritional value of soft-shell crabs.

## 5. Conclusions

This study demonstrates that different live-preservation methods influence the shell hardening process in soft-shell crabs. Both the ICE and LTA treatments significantly delayed shell hardening, with shells remaining at the soft-paper stage even 48 h after molting. This delay appears to be mediated through transcriptional regulation of key genes involved in calcium signaling and cellular remodeling pathways, including autophagy and lysosome function. Flavor analysis revealed that the ICE method enhanced umami by increasing levels of Asp, IMP, and AMP, and improved sweetness through elevated Ala and Gly, while simultaneously reducing bitterness by lowering BAA content. In contrast, the LTA method reduced both umami and bitterness but did not improve sweetness. Together, these results indicate that ice-chilled storage is the preferred method for live preservation of soft-shell crabs, as it effectively delays shell hardening while improving key flavor attributes of the hepatopancreas. Although the tactile scale is currently recognized as the practical standard for assessing soft-shell hardness, its subjective nature represents a significant limitation. Furthermore, the molecular mechanisms underlying flavor changes remain unknown. Future studies should focus on elucidating the specific mechanisms responsible for these changes.

## Figures and Tables

**Table 1:** Effects of different live-preservation methods on the contents of free amino acids (FAAs) in the hepatopancreas of soft-shell crabs.

FAA	Mean ± SD (mg/100 g)			Taste Attribute	Taste Threshold (mg/100 mL)	TAV		
	CK48	ICE48	LTA48			CK48	ICE48	LTA48
_L_-Glu	143.25 ± 13.05 ^a^	137.91 ± 6.66 ^a^	81.8 ± 3.25 ^b^	Umami	30	4.77	4.6	2.73
_L_-Asp	25.68 ± 2.58 ^b^	75.15 ± 4.72 ^a^	71.03 ± 3.12 ^a^	Umami	100	0.26	0.75	0.71
_L_-Ala	249.76 ± 20.24 ^c^	540.15 ± 30.62 ^a^	328.37 ± 17.31 ^b^	Sweet	60	4.16	9	5.47
Gly	119.01 ± 3.26 ^b^	137.87 ± 6.1 ^a^	117.21 ± 5.15 ^b^	Sweet	130	0.92	1.06	0.9
_L_-Pro	79.14 ± 3.9 ^a^	18.19 ± 3.23 ^b^	18.08 ± 0.62 ^b^	Sweet	300	0.26	0.06	0.06
_L_-Ser	49.69 ± 9.29 ^a^	10.76 ± 0.86 ^b^	8.39 ± 1.12 ^b^	Sweet	150	0.33	0.07	0.06
_L_-Thr	46.48 ± 8.52 ^a^	12.04 ± 1.96 ^b^	9.64 ± 1.11 ^b^	Sweet	260	0.18	0.05	0.04
_L_-Arg	426.38 ± 46.84 ^a^	387.02 ± 49.04 ^a^	273.57 ± 17.81 ^b^	Bitter	50	8.53	7.74	5.47
_L_-Lys	117.89 ± 5.73 ^a^	69.67 ± 7.45 ^c^	97.79 ± 2.71 ^b^	Bitter	50	2.36	1.39	1.96
_L_-Leu	112.07 ± 6.65 ^a^	19.68 ± 2.57 ^c^	26.76 ± 3.5 ^b^	Bitter	190	0.59	0.1	0.14
_L_-Phe	105.88 ± 2.46 ^a^	14.66 ± 2.34 ^c^	25.87 ± 1.92 ^b^	Bitter	90	1.18	0.16	0.29
_L_-Val	77.46 ± 2.46 ^a^	14.11 ± 2.55 ^c^	19.96 ± 1.45 ^b^	Bitter	40	1.94	0.35	0.5
_L_-Ile	69.63 ± 3.79 ^a^	9.5 ± 1.28 ^b^	10.75 ± 1.18 ^b^	Bitter	90	0.77	0.11	0.12
_L_-Tyr	63.24 ± 5.12 ^a^	20.18 ± 1.29 ^c^	36.27 ± 2.15 ^b^	Bitter				
_L_-His	47.84 ± 8.14 ^a^	11.98 ± 1.58 ^b^	16.67 ± 0.46 ^b^	Bitter	20	2.39	0.6	0.83
_D/L_-Trp	35.16 ± 4.59 ^a^	12.88 ± 1.87 ^c^	26.08 ± 2.34 ^b^	Bitter	200	0.18	0.06	0.13
_L_-Met	34.91 ± 3.33 ^a^	7.91 ± 0.47 ^c^	13.66 ± 0.5 ^b^	Bitter	30	1.16	0.26	0.46
_L_-Gln	107.04 ± 8.11 ^a^	46.74 ± 2.72 ^b^	32.52 ± 1.61 ^c^	Tasteless				
_L_-Asn	39.61 ± 8.54 ^a^	3.94 ± 0.33 ^b^	7.04 ± 0.5 ^b^	Tasteless				
_L_-Cys	7.54 ± 0.37 ^a^	5.04 ± 0.2 ^b^	4.85 ± 1.1 ^b^	Tasteless				
EAA	553 ± 18.22 ^a^	148.41 ± 6.47 ^c^	220.86 ± 10.53 ^b^					
UAA	168.92 ± 14.44 ^b^	213.06 ± 9.05 ^a^	152.83 ± 3.42 ^c^					
SAA	544.08 ± 21.9 ^b^	719.01 ± 34.14 ^a^	481.67 ± 18.55 ^c^					
BAA	1090.45 ± 68.67 ^a^	567.58 ± 53.73 ^b^	547.36 ± 21.23 ^b^					
TAA	1957.65 ± 96.63 ^a^	1555.35 ± 78.26 ^b^	1226.27 ± 16.81 ^c^					

Values are mean ± SD (*n* = 6). Different letters in the same row denote significant differences between groups (*p* < 0.05, Kruskal–Wallis test with Benjamini–Hochberg correction). Glu, glutamate; Asp, aspartate; Gly, glycine; Ala, alanine; Pro, proline; Thr, Threonine; Ser, serine; Arg, arginine; Lys, lysine; His, histidine; Tyr, tyrosine; Leu, leucine; Phe, phenylalanine; Val, valine; Met, methionine; Ile, isoleucine; Trp, tryptophan; Gln, glutamine; Asn, asparagine; Cys, cysteine; UAAs, Umami amino acids; SAAs, sweet amino acids; BAAs, bitter amino acids; EAAs, essential amino acids; TAAs, total free amino acids; TAV, taste activity value.

**Table 2:** Effects of different live-preservation methods on the contents of flavor nucleotides in the hepatopancreas of soft-shell crabs.

Nucleotide	Mean ± SD ^a^ (mg/100 g)			Taste Threshold (mg/100 mL)	TAV		
	CK48	ICE48	LTA48		CK48	ICE48	LTA48
IMP	91.79 ± 6.08 ^c^	233.12 ± 13.66 ^a^	106.32 ± 3.13 ^b^	25	3.67	9.32	4.25
AMP	43.22 ± 3.95 ^c^	53.45 ± 1.97 ^b^	122.79 ± 5.27 ^a^	50	0.86	1.07	2.46
GMP	9.46 ± 1.62 ^b^	11.81 ± 1.61 ^a^	10.24 ± 0.33 ^ab^	12.5	0.76	0.94	0.82
Total	144.46 ± 7.3 ^c^	298.38 ± 14.5 ^a^	239.35 ± 5.17 ^b^				

Values are mean ± SD (*n* = 6). Different letters in the same row denote significant differences (*p* < 0.05, Kruskal–Wallis test with Benjamini–Hochberg correction). Abbreviations: IMP, inosine monophosphate; AMP, adenosine monophosphate; GMP, guanosine monophosphate; TAV, taste activity value.

## Data Availability

The raw data were deposited in the National Center for Biotechnology Information (NCBI, USA) under an accession number PRJNA1366045.

## Supplementary Materials

The following supporting information can be downloaded at: https://www.mdpi.com/article/10.3390/foods15020344/s1, Table S1: Hub genes identified from co-expression network analysis and their significantly enriched KEGG pathways, Table S2: KEGG pathway enrichment analysis of shared DEGs (ICE48 vs. LTA48).
