# Supplementary material for: Effects of Different Live-Preservation Methods on Soft-Shell Hardening and Flavor Characteristics of the Mud Crab (Scylla paramamosain)
Source: Foods. 2026 Jan 17;15(2):344. doi: 10.3390/foods15020344 (PMC12841251; doi:10.3390/foods15020344)
Supplement: Supplementary file 1 [file foods-15-00344-s001.zip › foods-4063567-supplementary-Table S1.docx]

**Table S1** Hub genes identified from co-expression network analysis and their significantly enriched KEGG pathways.

| **Gene** | **Pathway_ description** |
| --- | --- |
| *LOC135094825* | cAMP signaling pathway |
|  | Neuroactive ligand-receptor interaction |
|  | Sphingolipid signaling pathway |
|  | Calcium signaling pathway |
|  | cGMP-PKG signaling pathway |
|  | Regulation of lipolysis in adipocytes |
|  | Vascular smooth muscle contraction |
| *LOC135094885* | Mitophagy - animal |
|  | Autophagy - animal |
| *LOC135095378* | Lysosome |
| *LOC135096170* | Ribosome biogenesis in eukaryotes |
| *LOC135100963* | PI3K-Akt signaling pathway |
|  | Cell adhesion molecules |
|  | ECM-receptor interaction |
|  | Regulation of actin cytoskeleton |
|  | Phagosome |
|  | Focal adhesion |
| *LOC135105560* | Ether lipid metabolism |
|  | Glycerophospholipid metabolism |
|  | cAMP signaling pathway |
|  | Phospholipase D signaling pathway |
|  | Sphingolipid signaling pathway |
|  | Ras signaling pathway |
|  | Endocytosis |
|  | GnRH signaling pathway |
|  | Fc gamma R-mediated phagocytosis |
|  | Glutamatergic synapse |
| *LOC135109239* | Hippo signaling pathway - fly |
| *LOC135111622* | SNARE interactions in vesicular transport |
| *LOC135112374* | Nucleocytoplasmic transport |
| *LOC135113540* | Folate biosynthesis |
| *LOC135115103* | Purine metabolism |
| *LOC135116433* | Lysine degradation |

The presented hub genes form a core interactome, filtered from high-confidence connections (|cor| > 0.8, q < 0.05) among key network nodes. Non-annotated or human disease-related genes were filtered out.
